# Excessive application of chemical fertilizer and organophosphorus pesticides induced total phosphorus loss from planting causing surface water eutrophication

**DOI:** 10.1038/s41598-021-02521-7

**Published:** 2021-11-26

**Authors:** Liyuan Liu, Xiangqun Zheng, Xiaocheng Wei, Zhang Kai, Yan Xu

**Affiliations:** 1 Institute of Plant Nutrition, Resources and Environment, Beijing Academy of Agriculture and Forestry Sciences, Beijing, 100089 China; 2 Agro-Environmental Protection Institute, Ministry of Agriculture and Rural Affairs, Tianjin, 300191 China

**Keywords:** Environmental chemistry, Environmental impact

## Abstract

Total phosphorus (TP) loss from planting was one of the resources causing agricultural non-point source pollution. It is significant to clarify the factors influencing TP loss, as well as explore the relationship between TP loss from planting and surface water eutrophication for making recommendations on the reduction of environmental pollution. In this study, the minimum and maximum of average TP loss was appeared in Qinghai and Shandong province with the TP loss of 7.7 × 10^2^ t and 7.5 × 10^3^ t from 2012 to 2014, respectively. The results of structural equation model (SEM) indicating that the effect of anthropogenic drivers on TP loss was more important than natural conditions due to the higher path coefficient of anthropogenic drivers (0.814) than that of natural conditions (0.130). For anthropogenic drivers, the path coefficients of usage of fertilizer and pesticides, which was often excessively applied in China, were 0.921 and 0.909, respectively causing they the two dominant factors affecting TP loss. Annual precipitation and relative humidity, which were belongs to natural conditions, increased TP loss by enhancing leaching and surface runoff. However, light duration could reduce TP loss by promoting crop growth and increasing TP absorption of crops, with a path coefficient of − 0.920. TP loss of each province in per unit area from planting was significantly correlated with TP concentration of its surface water (*p* < 0.05), suggesting that TP loss from planting was the main factor causing surface water eutrophication. This study targeted presented three proposals to reduce the TP loss from planting, including promotion of scientific fertilization technologies, restriction of organophosphorus pesticides, and popularization of water saving irrigation technologies. These findings as well as suggestions herein would provide direction for the reduction of TP loss from planting.

## Introduction

The intensification activities of human created considerable waste containing phosphorus^1^. Previous studies quantified the generation and recycling about phosphorus (P) waste in China, showing that the annual production of waste containing phosphorus increased sharply from 1900 to 2015^2^. In addition, numerous researchers demonstrated that the use and loss of phosphorus were all intensified greatly in the past years^3^, especially, planting was the largest source of phosphorus for most of the year^4^. The loss of phosphorus from agricultural fields was regarded as an important contributor for the eutrophication of water bodies in recent decades, especially for the situation where the phosphorus fertilizer applications was exceed the demand of the crop^5,6^. Excessive P in the soil can not only affect the quality of surface water and groundwater through runoff and infiltration respectively, but also cause soil compaction, thereby reducing cereal production^7–9^. Therefore, the loss of phosphorus from planting needs to be highly valued, as it is related to national water resources and food security to some extent.

Currently, most studies in China have focused on the crop yield and consumption related with phosphorus losses^10,11^. However, there is a lack of basic information about phosphorus loss in the planting industry on a large spatial scale, and the current status of phosphorus loss is not intuitively presented. As a result, everyone may not pay attention to the important problems that phosphorus loss will cause. Moreover, human disturbance has increased the loss of phosphorus from the land to the surface water, leading to the risks of phosphate rock depletion^12,13^. Some studies have begun to focus on implementing mitigation measures or changing external conditions to reduce phosphorus loss^14^. Measures such as, avoiding P applications on the soil with excessive fertilization would be effective control the source pressure, although this cannot correct the nutrient deficiency of the farm^15^. Studies noted that source control effort^8^, have found that the long-term realization of phosphorus reduction required a reduction in nutrient loads on agricultural soils, however, this would be affected by significant time lags due to hydrologic and biogeochemical factors^16^. The effects of climate and landuse on the phosphorus migration in the Jialing River Watershed, China during rainfall runoff was explored in the previous study^17^. There were also some studies that monitored the P loss based on a long-term fertilization experiment under simulated rainfall or changed slope gradient^18,19^. Vero et al. also evaluated the riskiness of runoff, management, and infrastructure factors on phosphorus loss from farmland to water, using expert opinion, of which runoff is high risk factor, but it may be difficult to alleviate compared with infrastructure^20^. However, within the scope of comprehensive factors, the main driving factors affecting the loss of phosphorus in China’s plantation industry have not yet been obtained, thus the loss of phosphorus cannot be accurately reduced, which will be the next focus.

We need to fully understand the loss of phosphorus in China's planting industry under the tempo-spatial scale and explore which factors are affected P loss and how this P loss can be avoided. In this study, we investigated the total phosphorus loss in planting industry of China from 2012 to 2014. The objectives of this research are as follows: (1) to understand the differences of phosphorus loss in China's planting industry at the temporal and spatial scales and influencing factors; (2) to clarify the contribution of TP loss from planting to the concentration of TP in surface water; and (3) to put forward how China should reduce phosphorus loss from the planting. This study will provide the basic data and theoretical support for enhancing phosphorus utilization and improving agricultural non-point source pollution.

## Materials and methods

The TP loss from 2012 to 2014 of China was collected from China Environmental Statistics Yearbook. The average TP concentration of surface water for each province was measured from state-controlled monitoring points varying from 12 to 386 points for each province. We searched the National Statistics Bureau of China for the natural factors and anthropogenic drivers. In the present study, the natural factors affecting TP loss included annual sunshine hours, annual precipitation, relative humidity, soil bulk density and soil cation exchange capacity. While, the anthropogenic drivers consist of agronomic measurements, which included fertilizer usage, pesticides usage and irrigation amount, and economic level which included gross domestic product (GDP) and per capita income of rural residents (PCIRR).

A structural equation model (SEM) was applied to quantify the contributions of each natural conditions and anthropogenic drivers on the TP loss from planting. SEM could analysis correlation among variables including potential variables, which could not be measured directly^21^. The system of SEM was shown in Table 1. SmartPLS 3.0 for windows was applied to calculate the path coefficient between two variables as shown in the Fig. 2. The linear correlation analysis between TP loss of each province in per unit area from planting and TP concentration of surface water was performed by Origin 9.

**Table 1:** Variable systems of TP loss, natural conditions and anthropogenic drivers established for SEM.

Variable	Potential variable	Observed variable
External potential variable	Natural conditions	Relative humidity
		Annual precipitation
		Soil cation exchange capacity
		Soil bulk density
		Annual sunshine hours
	Anthropogenic drivers	GDP
		PCIRR
		Fertilizers usage
		Pesticides usage
		Irrigation amount
Intrinsic potential variable	TP loss	The amount of TP loss from planting

## Results and discussion

### TP loss and the ratio of TP loss to sown area for China’s planting from 2012 to 2014

**Figure 1:**
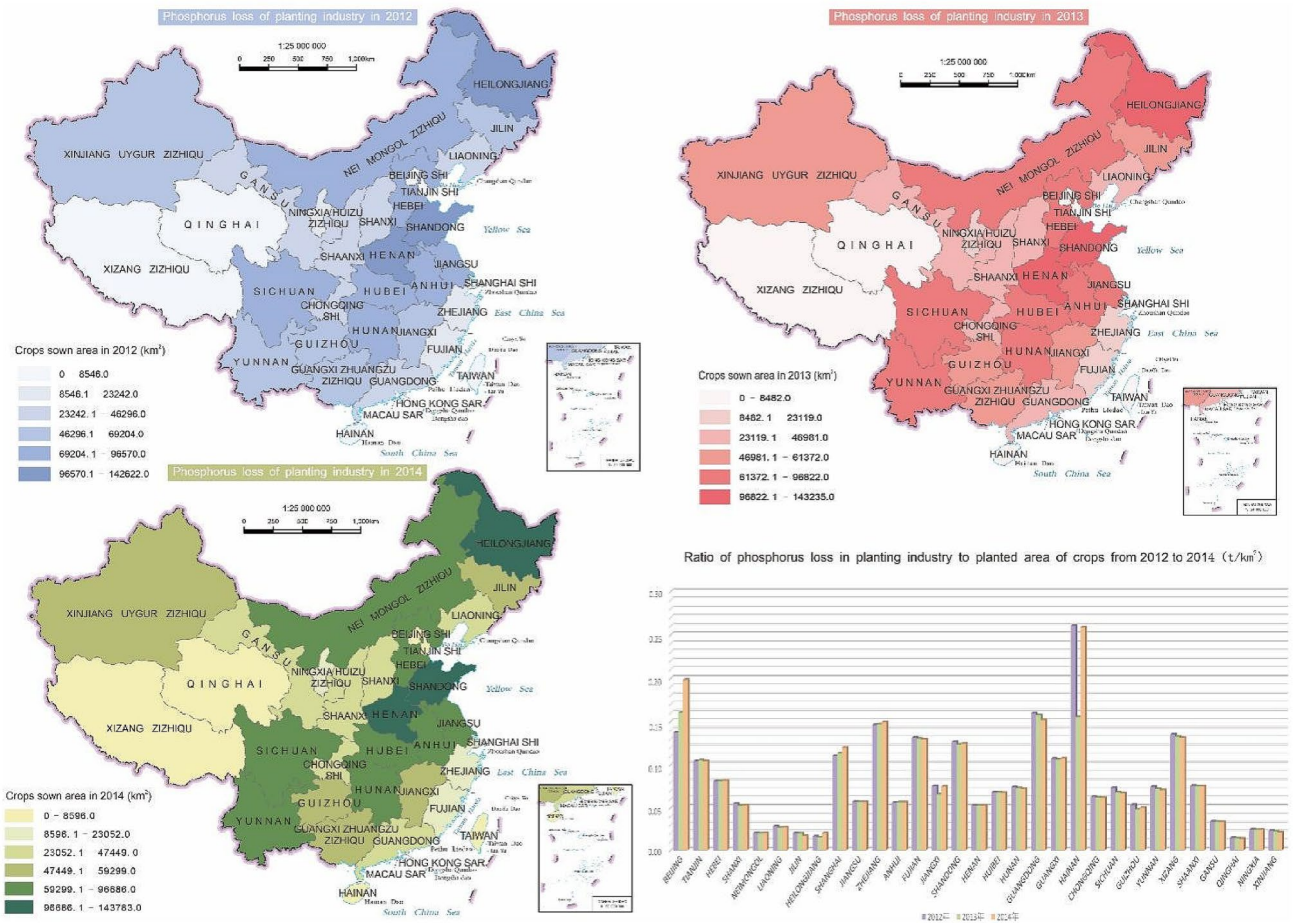
TP loss, crop sown area and the ratio of TP loss to sown area China from 2012 to 2014.

As shown in the Fig. 1, the TP loss and crop sown area of each province in China from 2012 to 2014 were relatively stable. From 2012 to 2014, the average level of national TP loss was 1.08 × 10^5^ t, in range from 7.7 × 10^2^ t to 1.3 × 10^4^ t for each province as shown in the Fig. 1. The highest TP loss was appeared in Shandong province which was followed by Henan and Guangdong province with an average TP loss of 7.5 × 10^3^ t and 7.3 × 10^3^ t, respectively. While Qinghai province, Ningxia Hui Autonomous Region and Tibet Autonomous Region was the three lowest TP loss provinces with 76.67 t, 299.67 t, and 329.13 t, respectively. Henan, Heilongjiang and Shandong provinces was the top three provinces of maximum sown area with 1.43 × 10^5^, 1.22 × 10^5^, and 1.10 × 10^5^ km^2^ respectively as shown in the Fig. 1 which was generated by CorelDraw 2019. The vegetable planting area of Shandong province was highest in northern China with more than 2000 km^2^. The study of Yang et al. indicated that the vegetable fertilizer usage was more than twice than that of field crops, which increase TP loss of Shandong Province^22^. The sown area of Qinghai province, Ningxia Hui Autonomous Region and Tibet Autonomous Region was only 5.5 × 10^3^, 1.3 × 10^4^ and 2.5 × 10^3^ km^2^, respectively. Even though the sown area of Qinghai province was higher than that of Tibet Autonomous Region, the fertilizer application amount was lower than that of Tibet Autonomous Region (Supplementary Fig. S1), which might be related to the situation of vigorously promotion of chemical fertilizer reduction policies in Qinghai province^23^. Lower fertilizer application rate of per area could increase fertilizer use efficiency and reduce TP loss because of the limited absorption of phosphorus by crops from soil^24^.

**Figure 2:**
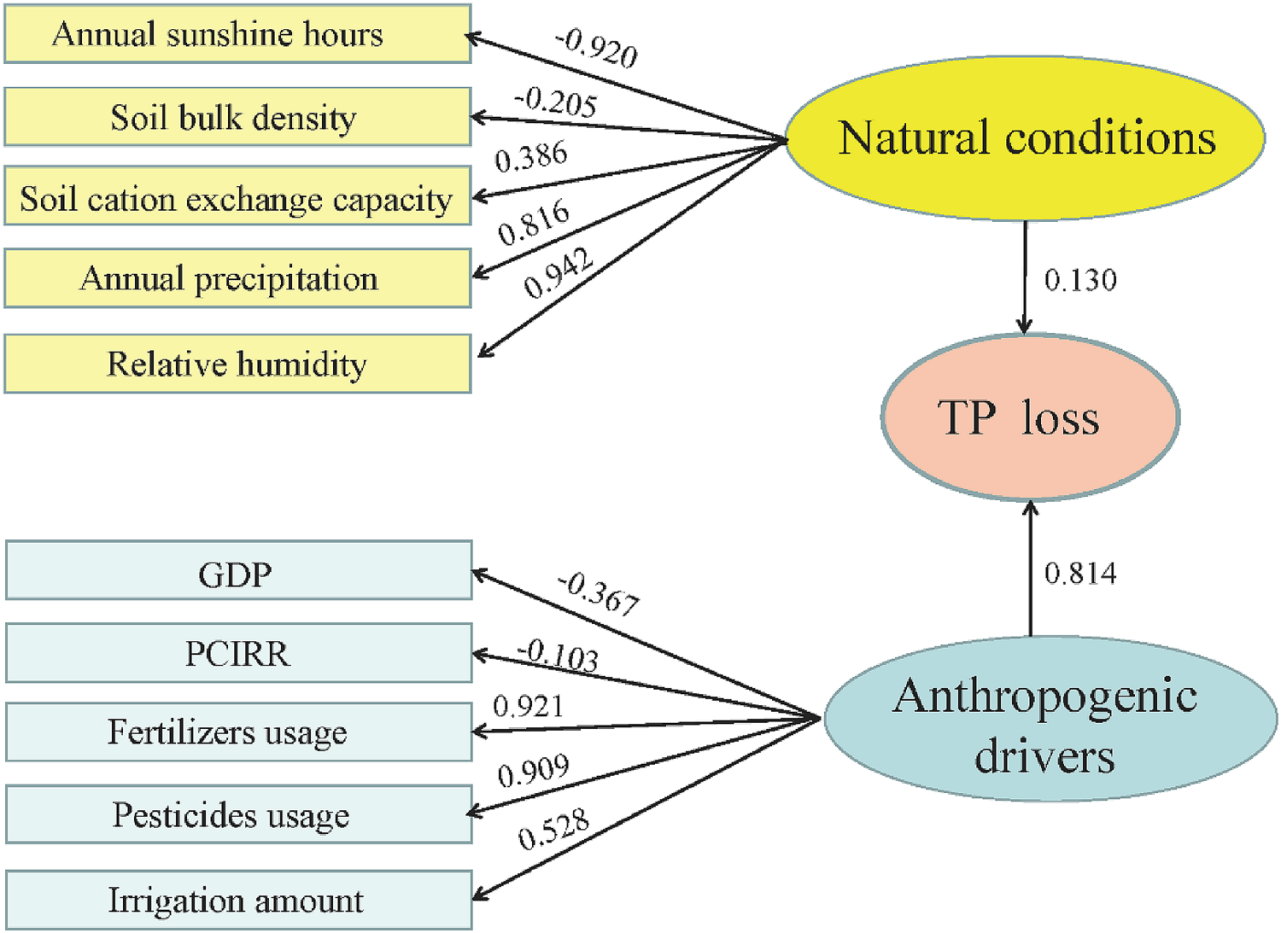
Structural equation model (SEM) illustrating the direct and indirect effects of natural conditions and anthropogenic drivers on TP Loss.

The variation of the ratio of TP loss to sown area for each province from 2012 to 2014 was shown in the Fig. 2. Average from 2012 to 2014, the ratio of TP loss to sown area of Hainan province was highest with the value of 0.22 t km^−2^, which induced by the highest fertilizer application amount of each sown area with 54.24 t km^−2^ (Supplementary Fig. S1). Comparely, the fertilizer application amount of each sown area in Qinghai province was lowest with 15.62 t km^−2^, and the ratio of TP loss to sown area was also lowest with the value of 0.014 t km^−2^. Previous study has demonstrated that TP loss was significantly positive correlated with phosphate fertilizer application per unit area^25^_._ In addition, average annual precipitation of Hainan and Qinghai province were approximately 1600 mm and 300 mm, respectively. Greater rate of precipitation could generate higher runoff amount, thereby increasing TP loss^26^, which was consistent with our findings.

### The influences of natural conditions on TP loss

In order to evaluate the discriminate influences of natural conditions and anthropogenic drivers on TP loss, SEM was constructed as shown in the Fig. 2. The results indicated that the effect of anthropogenic drivers on TP loss was more important than natural conditions due to the higher path coefficient of anthropogenic drivers (0.814) than that of natural conditions (0.130). The study of Liu et al., which concerned the driving forces of total nitrogen loss from crop production, also demonstrated anthropogenic drivers played a more important role on total nitrogen loss than natural conditions^27^.

The impacts of natural conditions on TP loss illustrated that annual precipitation, relative humidity, and light duration were the main three drivers influencing TP loss with path coefficients of 0.816, 0.942 and − 0.920 separately. It was well known that relative humidity was significantly affected by annual precipitation. Previous studies had demonstrated that leaching and surface runoff were the main two pathways of TP loss in farmland, which were closely related to precipitation^28,29^. The study of Tian et al., which concentrated on the relationship between precipitation and runoff based on the data of 18 hydrological stations, found that runoff coefficient was 24% higher in non-drought years than its in drought years^30^. Consequently, annual precipitation and relative humidity was positively related to the TP loss. However, the path coefficient of light duration was observed at − 0.920, suggesting that the increase light duration would reduce soil TP loss. Light was regarded as one of most important environmental factors influencing crop growth^31^. Good crop growth and development model would be built with adequate light, and more phosphorus was absorbed from soil, thereby reducing TP loss. Moreover, the path coefficient of soil bulk density was − 0.205 which revealed that soil bulk density was negative related to TP loss. Soil bulk density was seemed as one index of soil physical properties, which was significantly negative related to soil porosity^32^. In general, if the bulk density was higher, soil porosity was usually lower, which could increase water movement resistance and decrease phosphorus leaching. As shown in the Fig. 2, the path coefficient of CEC was 0.386, showing the positive effects of CEC on TP loss. Inorganic phosphorus in soluble forms could aggregate with cations including Ca^2+^, Mg^2+^, Al^3+^, and Fe^3+^ and switched to insoluble forms^33^, which was not conducive to be absorbed by crops^34^, resulting in the increase of TP loss.

### Impacts of anthropogenic drivers on TP loss

The top three anthropogenic drivers affecting TP loss were fertilizer usage, pesticides usage, and irrigation amount which all promoted the loss of TP (Fig. 2). It is a common phenomenon that the farmers in China generally applied extravagant fertilizers to decrease the risk of production loss resulted from insufficient nutrients^35^. However, studies indicated that the seasonal utilization efficiency of phosphate fertilizer in China was less than 30%^36^. In line with previous research, fertilizer usage was positively impacted TP loss and the path coefficient of fertilizer usage was 0.921. Similarly, the path coefficient of pesticides usage was 0.909 indicating that pesticides usage was also positively related to TP loss. Organophosphorus pesticides were widely used to remove the threat of agricultural pests on crop^37^. In 2010, the consumption of organophosphorus pesticides in China was around 3.0 × 10^5^ t, making up more than 70% of the whole pesticides usage^38^. However, the excessive organophosphorus pesticides would enter waterbody including pond and river with irrigation, precipitation and leaching^39^, which caused the TP loss from planting. It had been proved that irrigation would increase the solubility of P and its subsequent loss to water^40^. Irrigation amount had a positive effect on TP loss regarding the path coefficient of 0.528 in the present study. Water-saving irrigation decrease TP loss from runoff and leaching by 24.7–57.4% and 21.0–25.3%, respectively^41^. Although many significant achievements have been obtained in water-saving irrigation, conventional flooding irrigation remained the major type of irrigation in China^42^. In general, the irrigation amount of conventional flooding irrigation was often large. Take solar greenhouse vegetable production as an example, the total irrigation amount was nearly 1000 mm for each growing season with 60–70 mm per irrigation term averagely^43,44^, which far exceeded the demands of crop and water-holding capacity of soil. Excessive irrigation would lead to a significant increase of leakage, resulting in an increase in TP losses. It was notable that both GDP and PCIRR, which have the path coefficients of − 0.367 and − 0.103 separately, were all negatively correlated with TP loss. Farmers living in the district with higher GDP and PCIRR were more willing to explore advanced machinery, techniques, and management measures to increase fertilizer efficiency^45^.

### Relationship between TP loss of Planting and TP concentration in surface water

**Figure 3:**
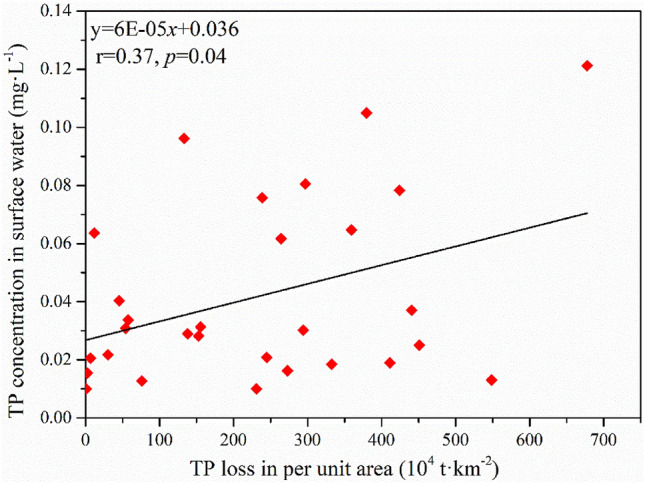
The linear regression between TP loss in per unit area of planting and TP concentration in surface water.

The linear correlation coefficient between TP loss in per unit area from planting and TP concentration in surface water of each province was 0.37 (*p* < 0.05) as shown in Fig. 3 which indicated that TP loss from planting have an influence on TP level in surface water. Extensive evidence indicated that phosphorus was one of the leading factors causing eutrophication of surface water^46–48^. Consequently, TP loss from planting is one of the main factors causing the surface water eutrophication. Previous studies have demonstrated that 67 lakes were eutrophic among the 131 major lakes in China^49^. Lake eutrophication has resulted in the degradation of the structure and function of lake ecosystem and huge economic loss^50^. Therefore, it is significant to make suggestions for the reduction of TP loss from planting for the protection of water resource.

### Suggestions for reduce TP loss

**Figure 4:**
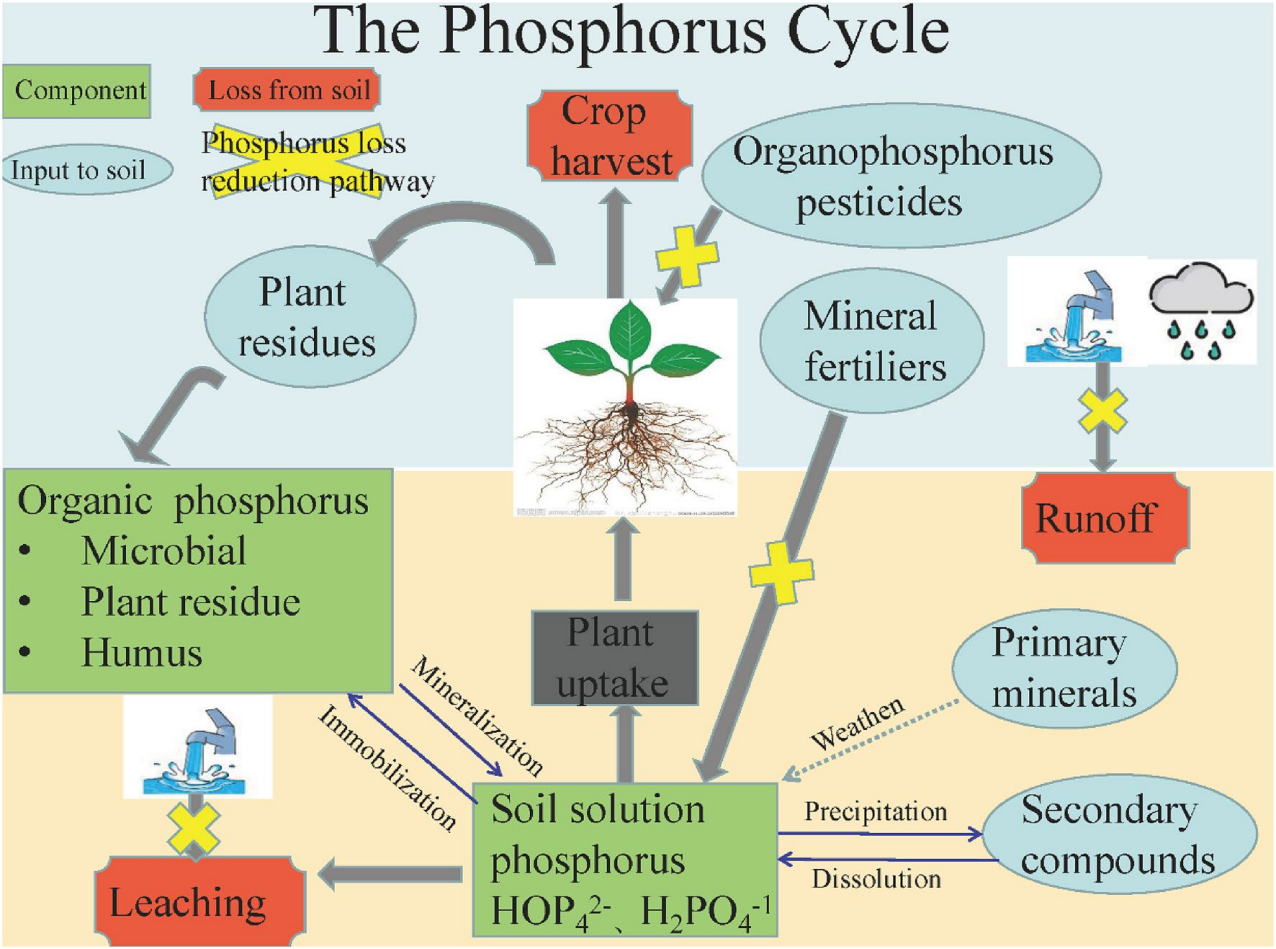
The concept of soil phosphorus cycle from planting and the priority control path for the reduction TP loss.

According to soil phosphorus cycle (Fig. 4) and above analysis, targeted suggestions for the reduction of TP loss from planting in China were as follow:

(i) Popularize scientific fertilization technologies including fertilizer reduction and precision fertilization. Large amounts of fertilizers were applied in order to get higher crop output in China. Previous study demonstrated that the amount of chemical fertilizer used in China was 2.5 and 2.6 times higher than that of EU and the United States respectively, which indicated that there was a great potential for the reduction of fertilizer application^51^. National key research and development project about chemical fertilizer reduction and efficiency increase had carried out in China. Balanced fertilization and organic–inorganic fertilizer combined application were regarded as two key technologies of chemical fertilizer reduction. In addition, soil type and soil fertility was heterogeneous in China^52^. Precision fertilization based on the results of soil testing and requirement of crops would can avoid waste and loss of the fertilizer due to blind fertilization.
(ii) Restrict the application of organophosphorus pesticides. Organophosphorus pesticide, which was widely used in the world, was an important factor for lake eutrophication due to the organic phosphorus loss^53^. The government of China has issued a number of policies to restrict the use of highly toxic organophosphorus pesticides including methamidophos, parathion and et al., since 2007^54^, such as “The prohibition of five highly toxic organophosphorus pesticides including methamidophos in agriculture, China (Ministry of Agriculture Announcement No. 322)”. However, the application amount of low toxic organophosphorus pesticides is still huge. It is meaningful to develop the substitute of organophosphorus pesticides to restrict the application of organophosphorus pesticides^55^.
(iii) Popularize water saving irrigation technologies including drip irrigation, furrow irrigation, shallow irrigation and deep storage and shallow-wet intermittent irrigation. For example, shallow irrigation and deep storage could decrease TP loss through runoff by 36.4%^56^. In addition, TP loss through runoff would decrease 10.0% under shallow-wet intermittent irrigation^57^.

## Conclusion

The maximum and minimum of the TP loss was observed in Shandong and Qinghai province, while the highest and lowest ratio of TP loss to sown area were appeared in Hainan province and Qinghai province, respectively. Anthropogenic drivers played a more important role on TP loss than natural conditions. For anthropogenic drivers, the usage of fertilizer and pesticides were demonstrated to be the two major factors influencing TP loss due to their excessive application. Annual precipitation and relative humidity increased TP loss by enhancing leaching and surface runoff. Contrarily, light duration could decrease TP loss, which mainly rely on promoting crop growth and increase TP absorption of crops. In addition, the linear correlation indicated that TP loss from planting might be one of the leading factors causing eutrophication of surface water. Popularization of scientific fertilization technologies, restriction of organophosphorus pesticides, and promotion of water saving irrigation technologies were supposed for decreasing TP loss from planting. Since the nature and extent of the agricultural non-point source pollution problem induced by TP loss in China has been uniquely and deeply developed, the three solutions mentioned above should to be implemented pertinently across China.

## Supplementary Information

Supplementary Information.
