# Supplementary figures and images for: Excessive application of chemical fertilizer and organophosphorus pesticides induced total phosphorus loss from planting causing surface water eutrophication
Source: Sci Rep. 2021 Nov 26;11:23015. doi: 10.1038/s41598-021-02521-7 (PMC8626514; doi:10.1038/s41598-021-02521-7)

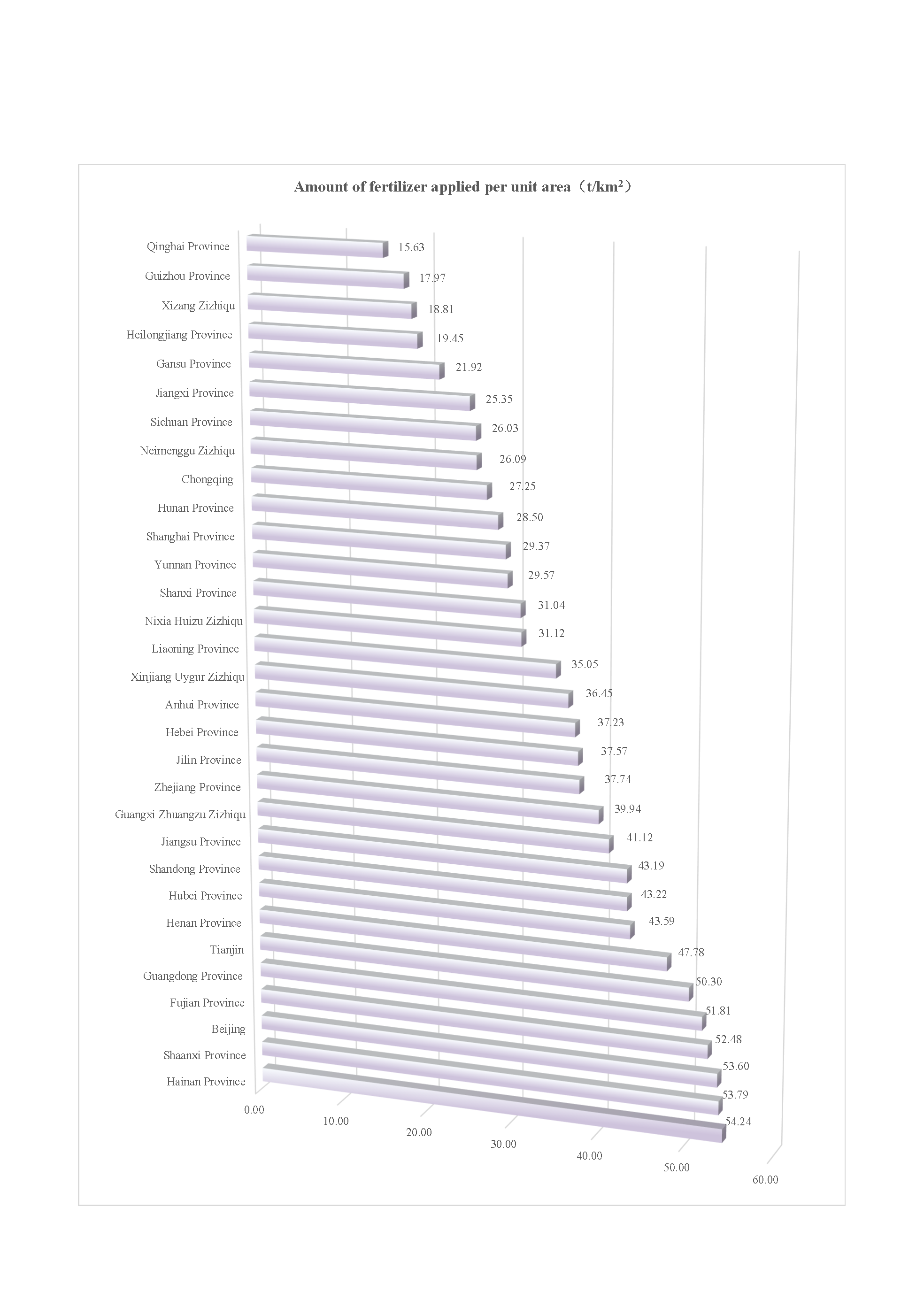

Supplement: Supplementary file 1 — Supplementary Information. [file 41598_2021_2521_MOESM1_ESM.tiff]
